# High Stability Thiol-Coated Gold Nanostars Monolayers with Photo-Thermal Antibacterial Activity and Wettability Control

**DOI:** 10.3390/nano9091288

**Published:** 2019-09-09

**Authors:** Davide Rovati, Benedetta Albini, Pietro Galinetto, Pietro Grisoli, Barbara Bassi, Piersandro Pallavicini, Giacomo Dacarro, Angelo Taglietti

**Affiliations:** 1 Department of Chemistry, University of Pavia, Viale Taramelli 12, 27100 Pavia, Italy; 2 Department of Physics, University of Pavia, Via Bassi 6, 27100 Pavia, Italy; 3 Department of Drug Sciences, University of Pavia, Viale Taramelli 14, 27100 Pavia, Italy

**Keywords:** antibacterial coatings, SERS, gold nanostars, photothermal effect, hyperthermia, thiols

## Abstract

The adhesion and proliferation of bacteria on abiotic surfaces pose challenges in both health care and industrial applications. Gold nanostars (GNSs) monolayers grafted on glass have demonstrated to exert antibacterial action due to their photo-thermal features. Here, these GNS layers were further functionalized using thiols monolayers, in order to impart different wettability to the surfaces and thus adding a feature that could help to fight bacterial proliferation. Thiol that has different functional groups was used and the thiol-protected surfaces were characterized by means of UV-vis spectroscopy, contact angles, SEM and surface enhanced Raman spectroscopy (SERS). We verified that (i) coating with the proper thiol allows us to impart high hydrophilicity or hydrophobicity to the surfaces (with contact angle values ranging from 10 to 120°); (ii) GNS monolayers are strongly stabilized by functionalization with thiols, with shelf stability increasing from a few weeks to more than three months and (iii) photo-thermal features and subsequent antibacterial effects caused by hyperthermia are not changed by thiols layers, allowing us to kill at least 99.99% of representative bacterial strains.

## 1. Introduction

Smart antibacterial surfaces are becoming the object of a recently boosted interest, as a response to the wide use of antimicrobial agents implicated in the pan-drug resistant bacteria emergency [1]. One popular possibility is given by the use of silver nanoparticles (AgNPs) grafted on bulk surfaces: Antibacterial surfaces are in this case based on the release of Ag^+^ cations having intrinsic antimicrobial features, with a wide range of action and limited possibilities of the generation of resistant strains [2,3,4,5]. Several other kinds of biocides can be used, by loading, embedding or grafting them to almost every kind of surface. Nevertheless, the general problem of biocidal releasing surfaces is always present: Surfaces tend to lose their efficiency as the antibacterial substances are released. Once the quantity of active molecules released becomes lower than the minimum inhibitory concentration (MIC), coatings will not be antibacterial anymore [6]. 

Noble metal nanoparticles, anyway, can be used to prepare antibacterial surfaces exploiting a completely different approach, i.e., the hyperthermia given by photo-thermal effects connected to their localized surface plasmon resonance (LSPR) absorption [7]. For example, gold nano-objects of anisotropic shape such as gold nanostars (GNSs) featuring two (or more) intense LSPR bands can produce neat hyperthermia in their surroundings as a result of thermal relaxation after laser irradiation, and this can be used to efficiently kill bacteria. This is especially valuable when the LSPR band falls in the near-IR “biological window” (NIR, 750–950 nm) in which biological fluids and tissues are semi-transparent. One can thus imagine applying this strategy to the treatment of possible infections on subcutaneous devices, if they are coated with this type of nanomaterials. In recent examples we started to investigate this strategy using self assembled monolayers (SAM) of GNS grafted on bulk glass surfaces [8]: Irradiation with a laser in the NIR allowed to exploit LSPR absorption of GNS, yielding a sensible hyperthermia that efficiently killed *Staphylococcus aureus* bacteria and eradicated the growing biofilm. Similar examples can be realized using silver nanoplates, conjugating LSPR based photo-thermal features with silver ion sustained release [9,10]. Alternatively, we succeeded in coating GNS monolayers with a thin layer of silica, and subsequently adding a further layer made of AgNP. In this way we combined the on-demand photo-switchable activity of GNS with the sustained biocidal release of silver ions, realizing surfaces on which both planktonic and surface attached bacteria could be efficiently killed [11]. The addition of the silica layer also offers a way to increase the stability of the GNS layers, avoiding contact with biomolecules, which, offering functional groups ready to bind to gold surfaces, could increase the risk of layer degradation or of proteins adhesion and subsequent ease of bacterial colonization. In all described cases, the success of the approach was pursued tailoring the LSPR absorption of the used nano-objects to a value close to 800 nm, in order to exploit laser irradiation in the biological window.

Anyway, another possibility to pursue these goals is to combine antimicrobial functions with surfaces having anti-adhesive properties [6]. It is known that strongly hydrophilic surfaces may render extremely difficult the adhesion of proteins and bacteria by forming a hydration layer [12], at the same time, super-hydrophobicity of surfaces was widely described as a tool that can be exploited for the same task [13]. Indeed bacteria are able to attach to various surfaces and multiply to form dense aggregates with a thickness ranging from a few nanometers to many centimeters, thus being able to avoid the initial adhesion may represent a true advantage in avoiding bacteria colonization and biofilm formation. 

**Scheme 1:**
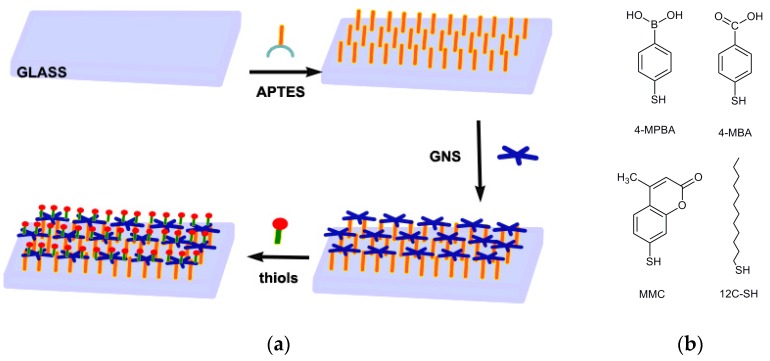
(**a**) The strategy pursued in this work used to obtain a gold nanostar (GNS) layer grafted on glass and functionalized with a layer of thiols; and (**b**) the thiols used in this work.

To our knowledge, the matching between anti-adhesive functions with photo-thermal antibacterial action is still largely unexplored. Our aim within this work is to start the investigation of the feasibility of this approach using the simple and efficient coating of the surfaces of GNS layers, grafted on the glass, whose formation is enabled by the creation of S–Au bonds (see Scheme 1); this strategy sounds logical after the pioneering contributions from Whitesides et al. [14,15,16]. We will thus show that strongly hydrophilic and hydrophobic features can be easily added to surfaces functionalized with GNS monolayers, and that such passivation with selected thiols for the desired function, can also greatly improve surfaces stability. Finally, we will show how this functionalization does not change the efficiency of the photo-thermally generated hyperthermia in killing bacteria.

## 2. Materials and Methods 

### 2.1. Materials

Gold(III) chloride trihydrate (~30 wt.% in HCl 99.99%), sodium borohydride (98%), L-ascorbic acid (AA) (≥ 99%), silver nitrate (99.8%), sodium citrate (≥ 99%), hydrochloric acid (≥ 37%), nitric acid (≥ 65%), sulfuric acid (95%), hydrogen peroxide (30 wt.%), ethanol (≥ 99.7%), toluen (≥ 99.7%)N-Dodecyl-N,N-dimethyl-3-ammoniun-1-propansulfunate (LSB; ≥ 99.7%), (3-aminopropyl)trimethoxysilane (APTES; ≥ 98.0%), L-glutathione, reduced (99%), 1-dodecanthiol (≥ 98%), 4-mercaptophenylboronic acid (≥ 99.7%), 4-mercaptobenzoic acid (99%) and 7-Mercapto-4-methylcoumarin (≥ 97%) were purchased from purchased from Sigma-Aldrich (St. Louis, MO, USA). Microscopy cover glass slides 21 mm × 6 mm were purchased from DEL Chimica (Milano, Italy).. All reagents were used as received. All the preparations were made with bidistilled water. 

Tryptone Soya Broth (TSB) and Tryptone Soya Agar (TSA) for bacteria culture were purchased from Oxoid, Basingstoke, Hampshire, UK. *S. aureus* ATCC 6538 and *Escherichia coli* ATCC 10536 bacterial strains were used.

### 2.2. Instruments

UV-Vis spectroscopy: UV-Vis-NIR absorption on functionalized slides was measured in air using a Varian Cary 50 UV/Vis spectrophotometer. The wavelength scan range was 300–1100 nm. The samples were placed in a special holder enabling transmission measurement of the same spot on the slide during all experimental stages.

Contact angle: Static contact angle determinations were made with a KSV CAM200 instrument, with the water sessile drop method.

Transmission electron microscopy (TEM): TEM images of the used GNS were taken on a Jeol JEM-1200 EX II instrument on 1:10 diluted GNSs solution, with a 10 μL sample dropped on copper grids (300 mesh) coated with a Parlodion membrane.

Scanning electron microscopy (SEM): SEM images were taken from a Tescan Mira XMU variable pressure Field Emission Scanning Electron Microscope—FEG SEM (Tescan USA Inc., Warrendale, PA, USA) located at the Arvedi Laboratory, CISRiC, Pavia. Slides were mounted onto aluminum stubs using double sided carbon adhesive tape and were then made electrically conductive by coating in vacuum with a thin layer of Pt/Pd (3–5 nm). Observations were made in backscattered electrons mode (BSE) at 30 kV and with an InBeam secondary electron detector for higher spatial resolution.

Raman and SERS: SERS spectra were acquired using the He–Ne 632.8 nm laser line as the excitation radiation. An Olympus microscope HS BX40 allowed for micro-sampling. The spectrometer was equipped with an 1800 g/mm grating and a cooled CCD camera was used as a photo-detector. The overall spectral resolution was about 1 cm^−1^. A 10× objective with a 2.8 mm spot diameter was used for colloidal samples (with Au concentrations of about 2.0 × 10^−4^ M, corresponding to 1.3 × 10^−9^ M of GNS) while spectra from cells were measured by means of a 50×objective with a 1.52 μm laser spot diameter. The focus depth was about 150 m and 15 μm for 10× and 50× respectively. Neutral filters with different optical density were used to irradiate the colloidal samples and the cells, leading to power density values of about 5 × 10^5^ W/cm^2^ and 8 × 10^4^ W/cm^2^, respectively. Typical integration times were about 20 s and the run was repeated five times. The estimation of the SERS enhancement factor was performed by comparing the SERS signal from the samples coated by three active thiols (MMC, 4-MBA and 4-MPBA) with the Raman signal from the same thiols diluted at 10^−2^ M in ethanol. The integrated intensities of the Raman modes considered for enhancement factor (EF) estimation were derived by the best-fitting procedure using Lorentzian curves as fitting functions. For the estimation of the SERS enhancement factor (EF) we used the equation EF = (I_SERS_ /I_RS_) × (N_RS_ /N_SERS_), where I_RS_ and I_SERS_ are the intensities of Raman and SERS peak of the same vibrational mode; N_RS_ and N_SERS_ are the numbers of molecules responsible for respectively Raman and SERS scattering. 10 μL of different molecules solution (3–10 M) was spread onto a blank glass slide with the diameter of 1 cm in order to evaluate I_RS_, while I_SERS_ values were derived by measuring SERS from glasses coated by 10^−5^ M solution of different thiols. The first requirement for a rigorous comparison between Raman and SERS signals is a fine control of the experimental conditions. We used the same laser wavelength and light power (632.8 nm and 1.5 × 10^5^ W/cm^2^), same integration times (20 s) and same objective magnification (50×). As already explained in our previous paper [17] to derive a reliable EF value it is important to evaluate the scattered volumes in the two experiments and considering that the SERS signal decreases dramatically with distance vanishing beyond 10–20 nm. In the present case the ratio between the scattering volumes in Raman and SERS experiments is approximately equal to 2 × 10^3^. On these bases we derived the EF values reported in the next section.

### 2.3. Methods

Glassware pre-treatment: All the glassware was usually pre-treated before use: A wash in aqua regia for 30 min, then washed and filled with bi-distilled water and ultrasonicated for 3 min before discarding the water. The bi-distilled water/ultrasound treatment was repeated three times. Then the glassware was dried in an oven for 1 h at 140 °C. Before APTES grafting, microscopy cover glass slides (21 mm × 26 mm) were treated with a piranha solution (3:1 sulfuric acid 95% and hydrogen peroxide 30 wt.%) for 30 min. Then the slides were washed in water under sonication for three minutes, three times. Then the glasses were dried in an oven for 1 h at 140 °C.

Synthesis of gold nanostars (GNSs): The synthesis was already reported in our previous papers [18,19]. Briefly, the seeds were prepared in a vial by adding 5.0 mL of LSB aqueous solution (0.2 M) and 5.0 mL of HAuCl_4_ aqueous solution (5 × 10^−4^ M). Then, 600 µL of an ice-cooled solution of NaBH_4_ in water (0.01 M) were added to the pale yellow solution obtained in the previous step. The as prepared brown-orange solution was gently hand-shaken for a couple of seconds; this seeds solution is efficient for the growth procedure of GNS for 180 min from preparation, if kept cold. The growth solution was prepared with 50 mL of LSB solution in water at the same concentration chosen for the seed solution (0.2 M), 1800 µL of AgNO_3_ in water (0.004 M), 50 mL of aqueous HAuCl_4_ (0.001 M) and 820 µL of an aqueous solution of L-ascorbic acid (0.078 M) mixed to obtain a colorless solution just after a few seconds of gentle mixing. Soon after ascorbic acid, 120 µL of seeds solution were added under a gentle shaking (2 s) to give a blue colloid, the intensity of which rapidly increased. The solution was allowed to react without agitation for 1 h. The colloidal suspensions were stored in the preparation flask, maintained in the dark and used within 7 days from preparation.

Functionalization of slides with APTES: The pre-treated cover glass were fully immersed in a solution of APTES 10% (v/v) in ethanol and allowed to react for 5 min at 60 °C, using a Hellendhal type glass staining jar. Typically, eight slides were coated in a single jar. The amino-modified glasses were washed three times under sonication with ethanol. After this step, the samples were gently dried under N_2_ flux.

Functionalization of slides with a SAM of GNS: APTES coated glass slides were fully immersed in a GNS colloidal suspension for 14 h, using a Hellendhal type glass staining jar. Typically, eight slides were coated in a single jar. After immersion, the slides were washed three times in water without sonication and carefully dried in N_2_ stream. Dried samples were stored in the dark in a desiccator.

Functionalization of GNS monolayers with a SAM of thiol: APTES-GNS coated glass slides were fully immersed in an ethanolic solution of the desired thiol, using a Hellendhal type glass staining jar, in a concentration range between 10^−3^ and 10^−5^ Mol/L. After 1 h of immersion, slides were carefully washed three times with ethanol and carefully dried under nitrogen stream.

Photothermal measurements: Thermograms were collected using a Thermocam FLIR E40 and using a L808P200 Thorlabs as a laser source (λ = 808 nm) using a spot of 1 cm of diameter, using a power ranging between 39 and 207 mW.

Thermal microbicidal tests: Antibacterial activity due to the photo-thermal effect was investigated against *Staphylococcus aureus* ATCC 6538 (Gram+) and *Escherichia coli* ATCC 10356 (Gram–). The microorganisms were grown overnight in Tryptone Soya Broth at 37 °C. Washed cells were resuspended in Dulbecco’s PBS and optical density (OD) was adjusted to 0.2 at 650 nm wavelength, corresponding approximately to 1 × 10^8^ colony forming units (CFU)/mL. One functionalized slide was cut in four sections of about 10 mm × 10 mm in order to be almost completely irradiated by the laser. A volume of 0.02 mL of bacterial suspension was deposited on two sections. For each pair of functionalized glasses, one was irradiated for 30 min whereas the other was not irradiated. After this time, the glass section covered with bacterial suspensions was suspended in 1 mL of sterile water, gently shaken and then water was suitable diluted in three different tubes: 1:100, 1:10000 and 1:100000. From each tube, 1 mL of suspension was taken and then cultured on Tryptone Soya Agar to count viable cells. The decimal-log reduction rate, i.e., the “thermal microbicidal effect”, ME_T_ was calculated with the following formula:

ME_T_ = log N_C_ − log N_T_, (1)

where N_C_ is the number of CFU/mL developed in contact (30′) with a control, not irradiated modified glass sample, and N_T_ the number of CFU/mL counted after exposure (30′) to modified glass samples and irradiation. 

A comparative microbicidal effect in the absence of irradiation was performed comparing the effect of all functionalized samples with the effect obtained with a “blank” glass slide, using an identical procedure and the same contact time, through the following formula:

ME = log N_C_ − log N_E_ (2)

where N_C_ is the number of CFU/mL developed in contact (30′) with the not irradiated and unmodified blank glass sample, and N_E_ the number of CFU/mL counted after exposure (30′) to the not irradiated modified glass samples.

## 3. Results and Discussion

### 3.1. Preparation of GNS Monolayer on Glass

APTES SAM are prepared on microscopy cover glass slides (21 mm × 26 mm) by means of a reported procedure, with a simple immersion of the slides in a Hellendhal type glass staining jar containing 10% APTES solution in methanol [4]. Success of silanization is evidenced by the contact angle (c.a.) measurements, showing a change from a value < 10° for the starting glass samples (piranha cleaned) to a value of 56 (5)°.

**Figure 1:**
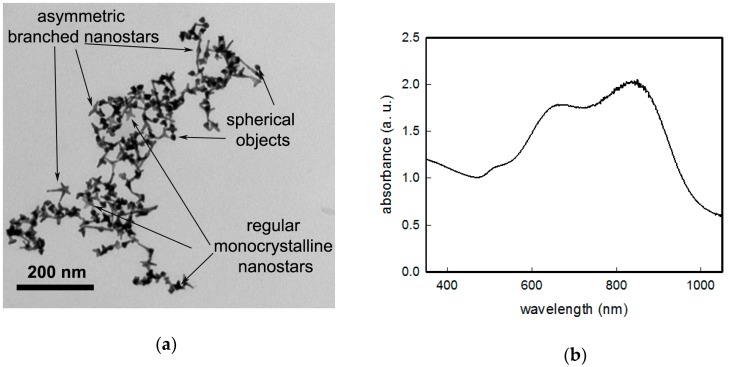
(**a**) A representative TEM of a standard preparation of GNS colloidal suspension; and (**b**) the UV-Vis-NIR spectrum of the same colloidal suspension.

As already reported, GNS are prepared using an seed-growth method in water, [18,19] the synthesis exploiting a zwitterionic surfactant, laurylsulfobetaine (LSB), which acts as the shape-directing agent, and the presence of ~10% (mol) Ag^+^. The product obtained using this approach consists of colloidal suspensions containing mainly two kinds of anisotropic objects: (i) Asymmetric branched gold nanostars and (ii) monocrystalline symmetric nanostars in a lower percentage. A representative TEM image is reported in Figure 1a. Composition of these mixtures, as well the dependence of shape features of the two kind of objects is strictly depending on the synthetic conditions, and this has been already described and fully characterized in our previous works [8,18] and will not be discussed here. Their extinction spectrum in colloidal suspension (see Figure 1 for a standard preparation) is dominated by the LSPR of the main (about 45% of objects) component, asymmetric branched gold nanostars, whose maximum can range in the 700–1150 nm interval as a function of synthetic parameters. A second LSPR band can be found in the 690–720 nm interval, owing to the more regular, monocrystalline symmetric nanostars (which represent about 35% of objects), with a central core diameter of 18 ± 4 nm and branches with a length of 12 ± 4 nm. A third, weak LSPR band close to 520 nm is due to the resonance of the spherical cores of the anisotropic objects and to spherical objects coming from seeds, which has undergone to a negligible developing of branches. It is important to notice that also slight and uncontrollable variations in synthetic conditions, which have been prepared as identical can yield slightly different shapes and populations of objects, resulting in colloidal suspensions, which may present small differences in position and intensities of LSPRs bands. Anyway, for this work we managed to obtain products having LSPR optical maxima for the highest wavelength band with a value always close to 850 (20) nm as reported in Figure 1b. 

Resulting GNS were coated by a double layer of surfactant (LSB), and this situation imparted a Z potential of ~−15 mV, as obtained by dynamic light scattering DLS measurements, while the pH of the colloidal suspension was close to 5.5. At this pH, the amino groups of APTES-terminated bulk glass samples were protonated, thus their immersion in the GNS suspension yielded the self-assembly of a GNS monolayer on glass samples, due to the electrostatic interactions between positively charged ammonium and negatively charged nano-objects. After 15 h of immersion of APTES silanized glass samples, samples were taken off and immersed in ethanol and then in water for washing, and finally treated with a flux of nitrogen.

Formation of the GNS SAM turned slides to an evident blue color. As already reported, SEM images show a homogeneous coating (*vide infra*) [11,17]. Typically, a LSPR blue shift of about 50–70 nm was observed moving from the colloidal suspension in water to the SAM on glass. The shift was due to the change of the local refractive index (n) on passing from water (n = 1.3339) to glass and air at the interface (n = 1.0003). 

The c.a. of the samples bringing the GNS monolayer was found to be 51(3)°.

As a result of the described blue-shift, the GNS monolayers samples were found to have LSPR maxima in the range 770–790 nm, a range due to the already mentioned variability of original colloidal suspensions of GNS used. Extinction of the long LSPR band was found to range between 0.20 and 0.25. Samples with spectral features outside of these ranges were discarded (less than 10% of prepared samples).

**Figure 2:**
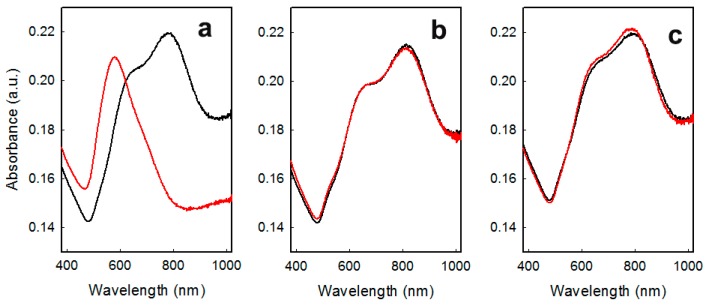
LSPR spectra of the: (**a**) GNS monolayer grafted on glass freshly prepared (black line) and after 2 months of storing in air (red line); (**b**) GNS monolayer grafted on glass coated with 4-MBA freshly prepared (black line) and after 3 months of storing in air (red line) and (**c**) GNS monolayer grafted on glass coated with 4-MBPA freshly prepared (black line) and after 3 months of storing in air (red line).

Samples were stable in air only for a few weeks, as evidenced by changes of the LSPR spectra (see Figure 2a), which suggests the almost total loss of anisotropic features after a couple of months. This is also evidenced by color changes from dark blue to wine red, suggesting that the gold nano-objects had become almost spherical. A similar behavior was observed after prolonged (> 5 h) immersion in water. This represents a striking difference in stability compared to the case of GNS grafted on glass by means of a (3-mercaptopropyl)trimethoxysilane (MPTS) layer we have described in the past [8]; this is not easily explainable, but could be attributed to the differences in the binding interactions, which are covalent for sulphur (S–Au bond) and electrostatic in the case of amines [17]. Recently, we have proposed silica coating as a tool to overcome this limited stability of APTES grafted GNS layers [11,17].

### 3.2. Coating the GNS Monolayers with a Monolayer of Thiol

Coating of the described GNS monolayers grafted on glass slides was performed by simple immersion of a set of slides (usually eight) in a Hellendhal type glass staining jar filled (40 cm^3^) with a 10^−5^ M thiol solution in EtOH for 1 h. The use of higher thiol concentrations or higher immersion times did not yield different values in the features of the coated monolayers (vide infra). After immersion and subsequent washing, samples could be stored and characterized.

The chosen thiols are reported in Table 1. 

In every case, a moderate red-shift in LSPR spectra was observed, especially for the long band, as a result of the change of the local refractive index experienced by GNS when thiols displace the labile double layer given by LSB. The extent of the red-shift is a function of the thiol features, and mean values observed are reported in Table 1, and in all cases brought the value of the LSPR maxima into the range of 790–830 nm. The LSPR spectra features were conserved upon coating, indicating that no changes in nano-object morphologies, shape and dimensions occurred during thiol coating. 

Measurement of static contact angles gave further evidence of success of thiol coating on the GNS grafted on glass. The value of c.a. moved from the original value of 51(3)° to values, which are peculiar of the functions brought on the used thiols, which are summarized in Table 1 (for some examples of contact angle measurements images see the supplementary material). From these data, a first, expected result can be clearly indicated: Modulation of wettability of gold nano-objects layers was obtained with this simple coating method, allowing to reach marked hydrophilicity imparted from 4-Mercaptophenylboronic acid (4-MPBA) and 4-Mercaptobenzoic acid (4-MBA), to intermediate values as in the case MMC, to the high hydrophobicity (120°) imparted by 1-dodecanthiol (12C-SH). 

Another interesting feature of these thiol-coated samples was their improved stability in time. As is evidenced by the spectra repeated after 3 months of storing in air without any particular care, no changes in LSPR spectra were observed for coated samples, as reported in Figure 2 for the coating with 4-MBA (Figure 2b) and 4-MPBA (Figure 2c). This behavior, observed for all thiols investigated, was strikingly different compared to what happened for samples aged in the absence of thiol coating for which prolonged storing in air produced a dramatic change in LSPR features, denouncing the loss of anisotropic features of grafted objects. This fact was immediately perceivable also by naked eye, by the color change described above [17]. 

**Figure 3:**
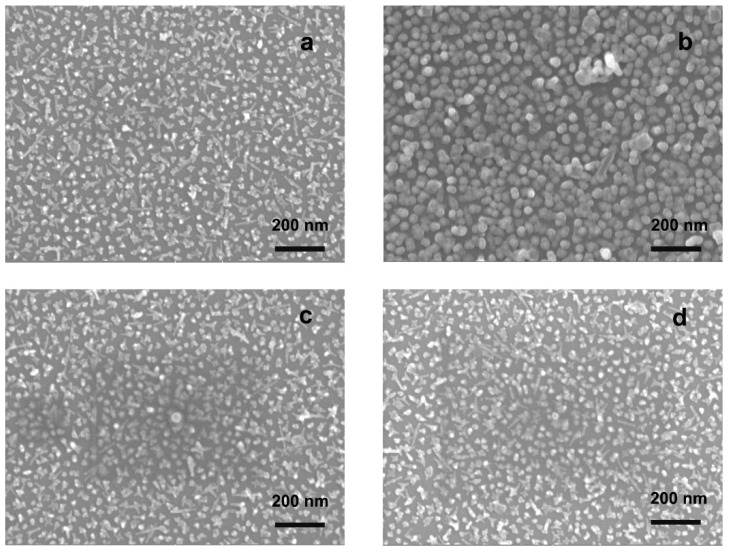
SEM images taken on: (**a**) the GNS monolayer grafted on glass freshly prepared; (**b**) the same after 2 months of storing in air, showing clear loss of anisotropy; (**c**) GNS monolayer grafted on glass coated with 4-MBA freshly prepared and (**d**) the same after 3 months of storing in air.

Figure 3 represents the SEM images obtained (a) for a freshly prepared and uncoated GNS layer; (b) for the same layer after 2 months of storage in air; (c) for a freshly prepared layer coated with 4-MBA and (d) and for the same layer after 3 months of aging.

As can be observed, the loss of the anisotropic features for the uncoated samples was confirmed, by the fact that only rounded objects could be observed on the surface after 2 months storing, as already suggested by the LSPR spectra. On the other side, the coated samples (whose SEM images were identical to the freshly prepared uncoated ones) showed no changes after a three month storing period. In this case the behavior was also anticipated by the UV-Vis spectra, as described above. SEM data for 4-MPBA coated GNS monolayers, freshly prepared and after 3 months of storing, are reported in the supplementary material (Figure S1).

The reason of this stabilization was not investigated further, even if it is not completely unexpected. The “native” GNS possesses a layer of LSB surfactant that is quite labile, as demonstrated by the fact that it is readily replaced by added thiols. Coating with thiols apparently freezes the anisotropic shape, avoiding object reorganization towards the more thermodynamically stable spheroidal shape, a fact that cannot be avoided in the presence of the labile surfactant layer, and which is observed in air and, much more faster, when immersed in water. As already cited, this instability is not observed when the same GNS are grafted on glass by means of a MPTS layer. This could be due to the difference in the nature of the bond features: The covalent S–Au bond versus the electrostatic nature of interaction between GNS and protonated amines in the APTES layer. A certain degree of mobility of gold nano-objects was already observed on APTES grafted gold nano-objects [19], which could contribute to instability. Nevertheless, as already stressed for objects grafted on glass using MPTS instead of APTES, the presence of thiols and of S–Au bonds seems to increase the stability of the nano-objects.

It is important to notice that the addition of thiols also offers the way to remove the surfactant molecules used for the seed-growth synthesis and stabilization of GNS: Surfactants may have an harmful effect when used in applications related to human health, as indeed it has been demonstrated that in some cases they are toxic for our cells [20]. Therefore their post-synthetic substitution with molecules, which are not toxic and/or more stably bound to nano-objects, should be considered a necessary improvement.

**Figure 4:**
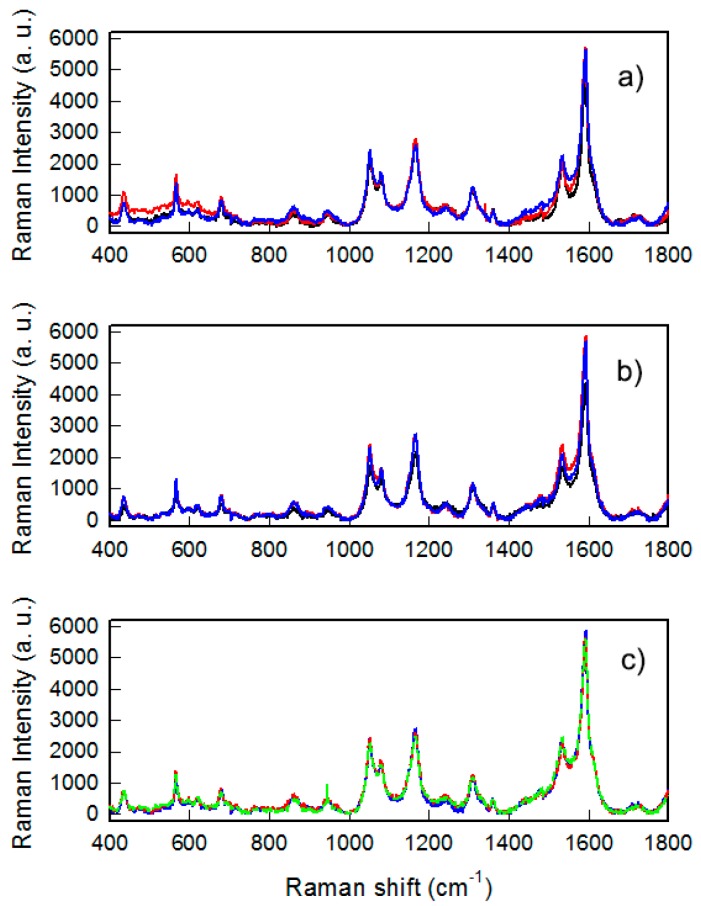
Surface enhanced Raman spectroscopy (SERS) spectra of GNS layers coated with MMC: (**a**) Taken on three different samples; (**b**) taken on three points of the same sample and (**c**) taken on three different samples prepared using coating solution with different concentrations: 10^−5^ (green line), 10^−4^ (blue line) and 10^−3^ (red line).

The coating by thiols was also characterized by SERS experiments. SERS enhancement of the usually extremely weak Raman signal allowed us to reveal very low amounts of molecules bound or adsorbed to a nano-structured metallic surface. The enhancement factor (EF) of a specific Raman signal and the spectral features of SERS spectra could give information about specific groups involved in bonds and/or specific geometries with which the molecules are bound or adsorbed [21]. We measured the spectra of the samples coated with three Raman active thiols: MMC, 4-MBA and 4-MPBA. The SERS spectra taken on different points of a single sample and on different samples for MMC are reported in Figure 4. As can be clearly noticed, spectra recorder from different points of the sample and from different samples were almost overlapped, with differences in peak intensities of about 10%, a value considered as a marker of highly reproducible SERS substrates [22]. In particular this is true for the signals in the range 1000–1800 cm^−1^ where fall the main Raman modes due to the in-plane C=C stretching of the lactone ring and to the modes inside the benzene ring [23]. These results indicate the success of the thiol coating because the MMC molecule at this amount can be seen only exploiting the SERS effect. In turn the data reveal both a high uniformity of the coating on the samples coupled to a high reproducibility of the preparations (Figure 4a,b).

Preparations were also repeated increasing the quantity of MMC in the coating solutions used from 10^−5^M to 10^−3^M (Figure 4c): No changes in the whole SERS yield were measured, indicating the fact that the conditions used for a standard coating procedure (40 mL of 10^−5^ solutions of thiols for eight slides) ensure the formation of a monolayer of thiols fully covering the available surface of GNS, and that increasing the thiol concentration did not produce any increase of thiol binding, as already suggested by LSPR shift and c.a. values determinations.

SERS experiments were repeated with 4-MBA and 4-MPBA. The spectra are reported in the supplementary material (Figures S2 and S3). For these thiols, SERS measurements also gave evidence that the coating was realized with success and that the coated substrates exhibited high reproducibility and homogeneity. As a completion of SERS characterization we calculated the enhancement factors for the three thiols. In all the cases we estimated the EF values by analyzing the Raman modes appearing in all the spectra around 1600 cm^−1^, because in these regions the SERS signal from thiols is not disturbed by other Raman/SERS signals and by unresolved background. As already reported above for MMC the Raman features around 1600 cm^−1^ originate from the carbon stretching inside lactone and benzene ring; similar origin, i.e., C–C motion in the benzene ring, can be attributed to the signal at 1590 cm^−1^ observed both for 4-MBA and 4-MBPA [24,25].

The EF values derived from the three thiols ranged from 5.4 × 10^6^ for MMC to 2.3 × 10^7^ for 4-MBA with the value for 4-MBPA being intermediate. Considering the non-resonant conditions, these values testified the good SERS performances of the nano-objects [26].

### 3.3. Photo-Thermal Dehavior

**Figure 5:**
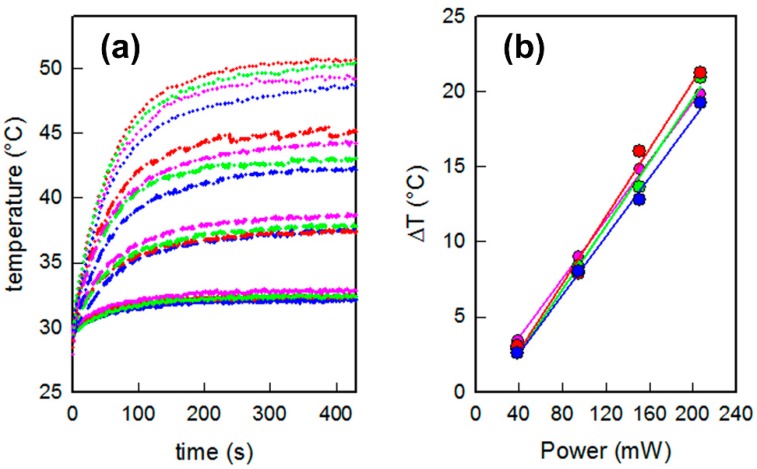
(**a**) Temperature versus time thermograms obtained for different samples under laser irradiation at 808 nm with different power samples: 39 mW (solid lines), 95 mW (long dashed lines), 151 mW (dash-dot-dash lines) and 207 mW (dotted lines); and (**b**) maximum temperature increase reached by each sample vs. applied laser power. Blue lines/symbols: Uncoated GNS layers; red lines/symbols: 4-MBA coated GNS layers; green lines/symbols: 4-MPBA coated GNS layers; pink lines/symbols: 12C-SH coated GNS layers.

The efficient photo-thermal properties of the GNS used in this work has been already studied on their colloidal suspensions, and when grafted on a SAM obtained with MPTS or APTES on glass [8,11,18]. Here we tested the photo-thermal behavior for the described samples, coming from the same preparation sets described and characterized so far, for the coatings with 4-MBA, 4-MPBA and C12-SH, which are the thiols giving the most interesting wettability values (i.e., the most hydrophilic and hydrophobic: The sample coated with MMC, having an intermediate c.a. value, were not investigated at this point). The slides were irradiated with continuous laser sources at 808 nm, using increasing power ranging from 39 to 207 mW (laser spot area = 0.785 cm^2^, irradiances 0.049–0.264 W/cm^2^). The temperature reached on the samples was measured vs. time using a thermocamera. The same steep ascending-plateau profiles were observed for all samples at every irradiance, as reported in Figure 5a. The maximum equilibrium temperature was reached in about 5 min, with ΔT increasing linearly from 3 to about 20 °C as a function of the applied laser power. Interestingly, no variations were observed in the relation between applied power and ΔT obtained on changing the thiol coating: The slopes of the lines interpolating the experimental ΔT vs. power data were almost the same for all samples, see Figure 5b. Thus, it was possible to conclude that laser irradiation of the GNS monolayers at 808 nm gave the same efficient photo-thermal conversion independently from the choice of the thiol used for coating, as expected from the observation that thiol coatings produced only negligible variations in the position and intensities of LSPR absorptions, which were only responsible for the photo-thermal efficiency.

The stability of the layers upon laser irradiation was controlled taking SERS spectra after 30 min of irradiation using the higher irradiance value, and comparing them with the spectra taken before irradiation. This was repeated three times for the samples coated with 4-MBA and 4-MPBA (as 12C-SH had no Raman features to be easily exploited with SERS): No changes in SERS patterns and intensities were perceivable, as can be observed in Figures S4 and S5. The same was observed when measuring LSPR features of the samples and their c.a. values. All these findings suggest that laser irradiations and the consequent hyperthermia did not produce any change in layers features.

### 3.4. Hyperthermia Based Antibacterial Effects on Planktonic Bacteria Cells

To test the effects of hyperthermia in eliminating Gram + as Gram – “planktonic” bacterial specimens (i.e., fluctuating, not yet organized in biofilm), samples of glass slides were opportunely cut in order to be completely covered by the laser spot while kept in contact with the desired bacteria suspensions, and were irradiated for 30 min with the described laser source at 808 nm, at an irradiance of 0.264 W/cm^2^. Strains of *E. coli* and *S. aureus* were chosen as representative of Gram – and Gram + bacteria, respectively. The “thermal microbicidal effect”, ME_T_, can be calculated using the formula ME_T_ = log N_C_ − log N_T_, where N_C_ is the number of CFU/mL developed after the contact with a functionalized glass sample in the absence of irradiation, taken as a control, and N_T_ the number of CFU/mL found in a sample kept in identical conditions but after exposure to the laser irradiation.

It is important to observe that in these conditions, the ME_T_ has to be ascribed only to the hyperthermia given by the photo-thermal effect, as any other influence from the features of the surfaces in the absence of irradiation is considered in the control count. Nevertheless, the so called, “microbicidal effect” (ME) measured in the absence of any irradiation, using the same contact time and calculated using a blank glass as a control, gave very low values (well below 1) in all cases. It is also important to stress that laser irradiation alone, in the absence of plasmonic objects exerting photo-thermal features, does not cause any harm to bacteria, as already observed [8,9,10,11]. Table 2 reports the ME_T_ obtained for freshly prepared samples of slides bearing uncoated GNS, and for samples coated with a monolayer of 4-MBA, of 4-MPBA and C12-SH.

As can be noticed, a strong microbicidal effect was obtained in all samples for the two bacteria strains investigated. In all cases the ME_T_ was higher than 4, a value corresponding to the elimination of at least 99.99% of bacteria and, specifically for *E. coli*, no bacteria at all were found after laser treatment. These results show that the laser treatment was able to eliminate almost completely the two investigated strains of planktonic bacteria cells after 30 min of irradiation at 0.264 W/cm^2^ (a value which is well below the considered safe for the exposure of skin [27]) at a wavelength that can be used in-vivo. Indeed, behavior of these surfaces in complex physiological environments, as well as their effect on healthy cells needs further investigation. In any case, the stability in physiological media and safety towards healthy cells has been already assessed in our previous papers for colloidal GNS coated with thiols like 7-Mercapto-4-methylcoumarin and PEG-thiols [28,29].

Of course, one can notice that the uncoated GNS layers (i.e., the samples which were not treated with the thiols and which conserve the original surfactant layer on the part of the GNS surface exposed to the environment) were already extremely active in killing planktonic bacteria with their photo-thermal features. Nevertheless their practical use was almost totally hampered by their instability, while, on the other side, the coating with thiols impart stability without altering photo-thermal efficiency in bacteria killing (see Figure S6 for a schematic illustration of the improved stability).

## 4. Conclusions

By means of the use of several techniques (UV-vis spectroscopy, contact angle measurements, SEM and Raman spectroscopy) we verified that coating with a monolayer of thiols could be easily obtained by means of immersion in a ethanolic solution of GNS monolayers grafted on glass, and that in this way it is easy to impart the desired hydrophilicity or hydrophobicity to the surfaces. Moreover, we found that the noble metal objects monolayers were strongly stabilized by this functionalization with thiols: after this passivation they were indefinitely stable in air and water solution, and also inert toward the heat generated by NIR laser irradiation. We also evidenced that addition of this layer of thiols was not changing their photo-thermal features and subsequent antibacterial effects connected to the generated hyperthermia. These findings demonstrated that this simple functionalization step, when applied to photo-thermally active plasmonic nano-objects grafted on the desired surfaces (for example those of a medical device, like catheter or a prosthetic medical implant or more generally of a nosocomial environment), can greatly improve their stability, and jointly add the desired surface wettability features without changing their antibacterial abilities towards planktonic bacteria. The next step of this work will consequently involve the study of cooperativity between wettability features and photo-thermal microbicidal effects when applied to sessile colonies of bacteria and biofilms, together with a complete investigation of the behavior of these nanomaterials in complex physiological environments (where protein binding to the thiol coated GNS surfaces could lead to adverse biological effects), and with the evaluation of possible toxicity to healthy cells in the human body.

## Figures and Tables

**Table 1:** Values for contact angles and for the shift of LSPR maxima upon thiol coating of GNS monolayers grafted on glass.

Thiol	c.a.	Δλ
//	51 (3)^1^	//
4-Mercaptophenylboronic acid (4-MPBA)	10 (3)	21 (4)^2^
4-Mercaptobenzoic acid (4-MBA)	18 (3)	30 (5)
7-mercapto-4-metilcoumarine (MMC)	34 (6)	48 (8)
1-dodecanthiol (12C-SH)	120 (10)	25 (2)

^1^ all c.a. data are calculated on the basis of at least two measures each on a minimum of four samples coming from different preparation batches; and ^2^ calculated on the differences from spectra of at least four different samples.

**Table 2:** Thermal microbicidal effects measured for GNS layers coated with different thiols.

	*Staphylococcus aureus*	*Escherichia coli*
Uncoated GNS	>5^1^	>5
4-MBA	4.5 (0.5)	>5
4-MPBA	4.5 (0.5)	>5
12C-SH	>5	>5

^1^ Values were obtained as the average of at least three experiments, uncertainties are given in parenthesis—a value higher than 5 indicates that no survived bacteria were found after the test.

## Supplementary Materials

The following are available online at https://www.mdpi.com/2079-4991/9/9/1288/s1, Figure S1: SEM images of sample coated with 4-MBPA freshly prepared and after 3 months of storing in air, Figure S2: SERS spectra of GNS layers coated with MBA, Figure S3: SERS spectra of GNS layers coated with MPBA, Figure S4 and S5: SERS spectra of GNS layers coated with MBA/MPBA before and after laser irradiation for 30′ at 808 nm for one and three cycles, Figure S6: schematic illustration of the improved stability, Figure S7: examples of images from contact angle measurements.
