# Supplementary material for: High Stability Thiol-Coated Gold Nanostars Monolayers with Photo-Thermal Antibacterial Activity and Wettability Control
Source: Nanomaterials (Basel). 2019 Sep 9;9(9):1288. doi: 10.3390/nano9091288 (PMC6781089; doi:10.3390/nano9091288)
Supplement: Supplementary file 1 [file nanomaterials-09-01288-s001.pdf]

Supplementary Material

High stability thiol-coated Gold Nanostars monolayers with photo-thermal antibacterial activity and wettability control.

Davide Rovati[1], Benedetta Albini[2], Pietro Galinetto[2], Pietro Grisoli[3], Barbara Bassi[1], Piersandro Pallavicini[1], Giacomo Dacarro[1], and Angelo Taglietti[1]*

[1] Department of Chemistry, University of Pavia, Viale Taramelli 12, 27100 Pavia , Italy; E-mail: angelo.taglietti@unipv.it

[2] Department of Physics, University of Pavia, Via Bassi 6, 27100 Pavia , Italy.

[3] Department of Drug Sciences, University of Pavia, Viale Taramelli 14, 27100 Pavia , Italy.

* Correspondence: angelo.taglietti@unipv.it; Tel.: +39-382-987342

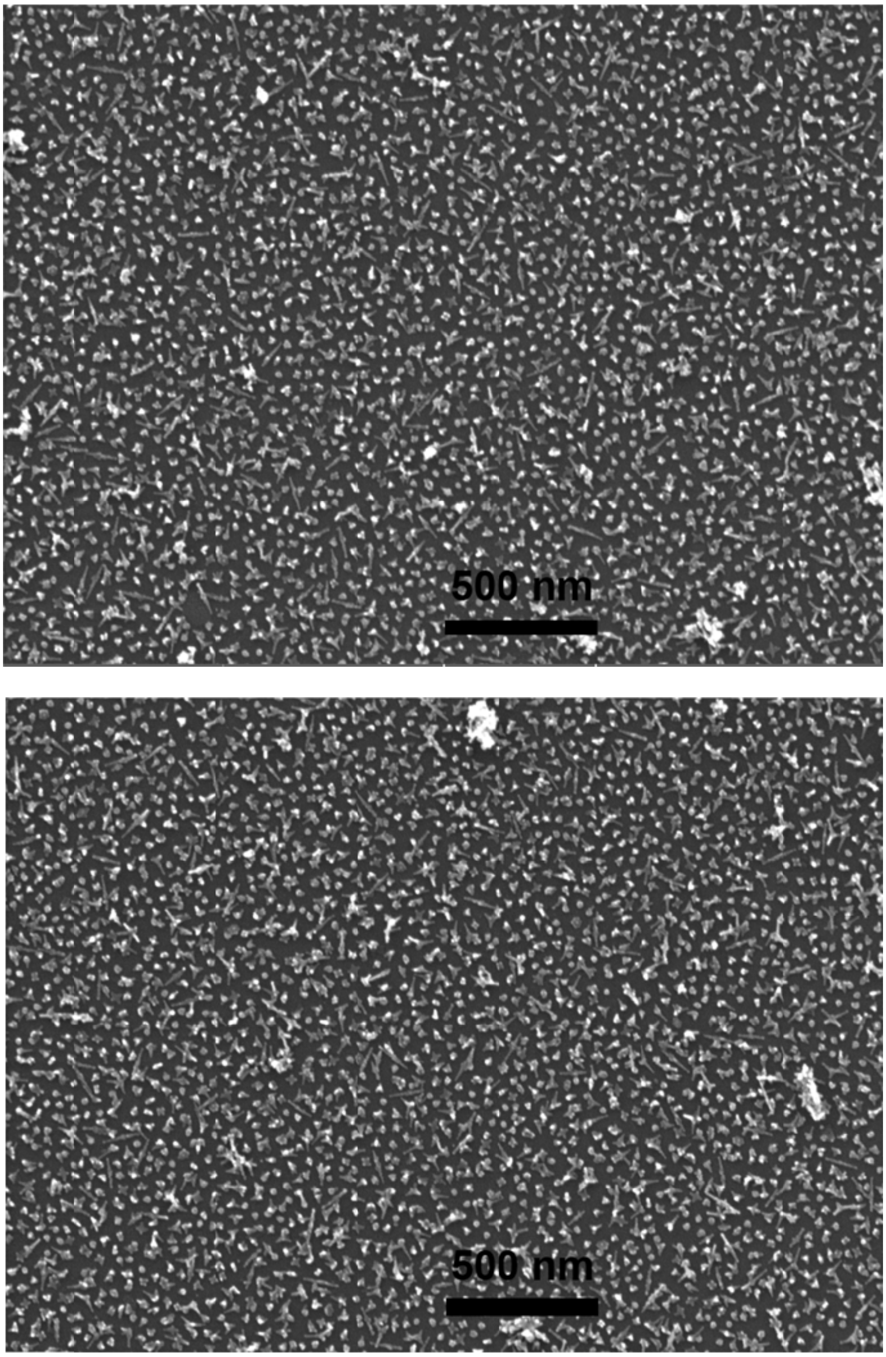

Figure S1. SEM images of samples coated with 4-MBPA freshly prepared (upper image) and after 3 months of storing in air (lower image)

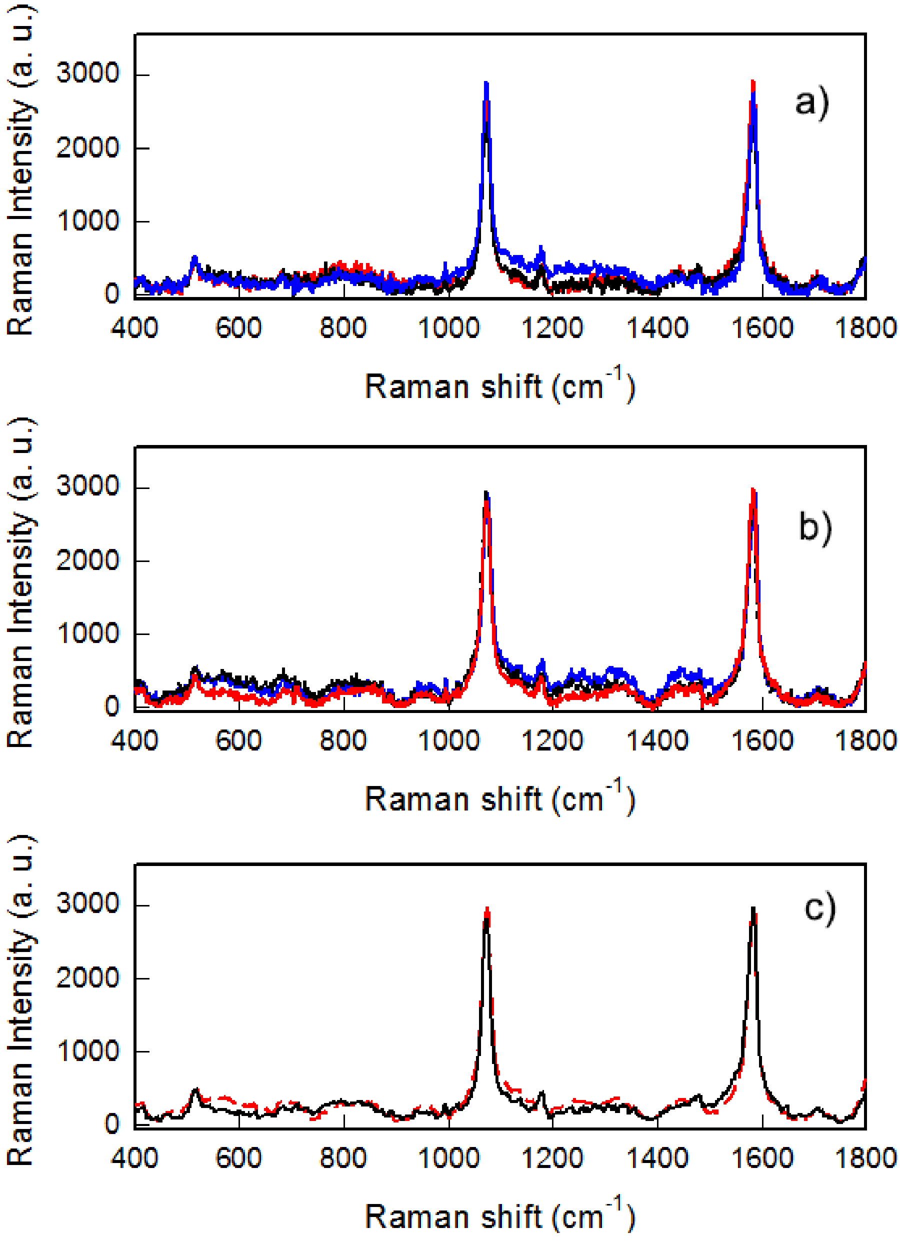

Figure S2. SERS spectra of GNS layers coated with MBA : (**a**) taken on three different samples; (**b**) taken on three points of the same sample; (**c**) taken on two different samples prepared using coating solution with different concentration: 10^{-5} (black line) 10^{-3} (red line).

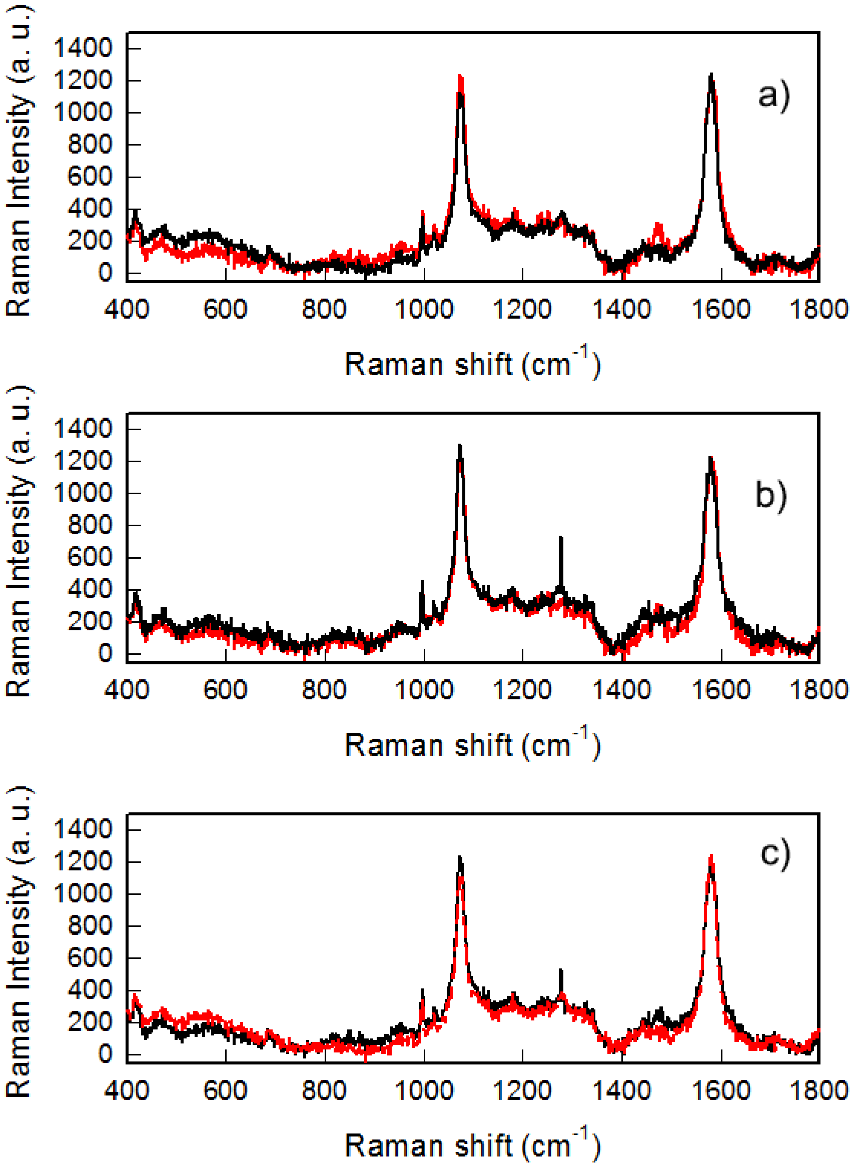

Figure S3. SERS spectra of GNS layers coated with MPBA : (**a**) taken on two different samples; (**b**) taken on two points of the same sample; (**c**) taken on two different samples prepared using coating solution with different concentration: 10^{-5} (black line) 10^{-3} (red line).

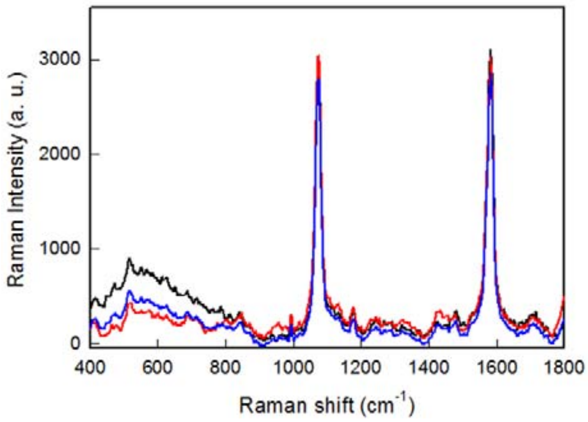

Figure S4. SERS spectra of GNS layers coated with MBA taken on the same sample before (red line), after one cycle (black line) and after three cycles (blue line) of 30' irradiation at 808 nm with irradiance 0.264 W/cm^2

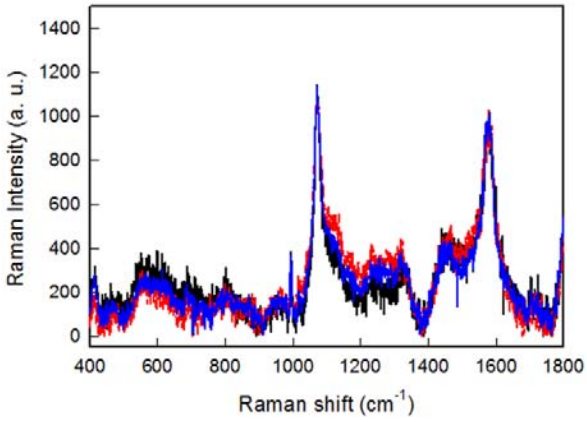

Figure S5. SERS spectra of GNS layers coated with MPBA taken on the same sample before (red line), after one cycle (black line) and after three cycles (blue line) of 30' irradiation at 808 nm with irradiance 0.264 W/cm^2

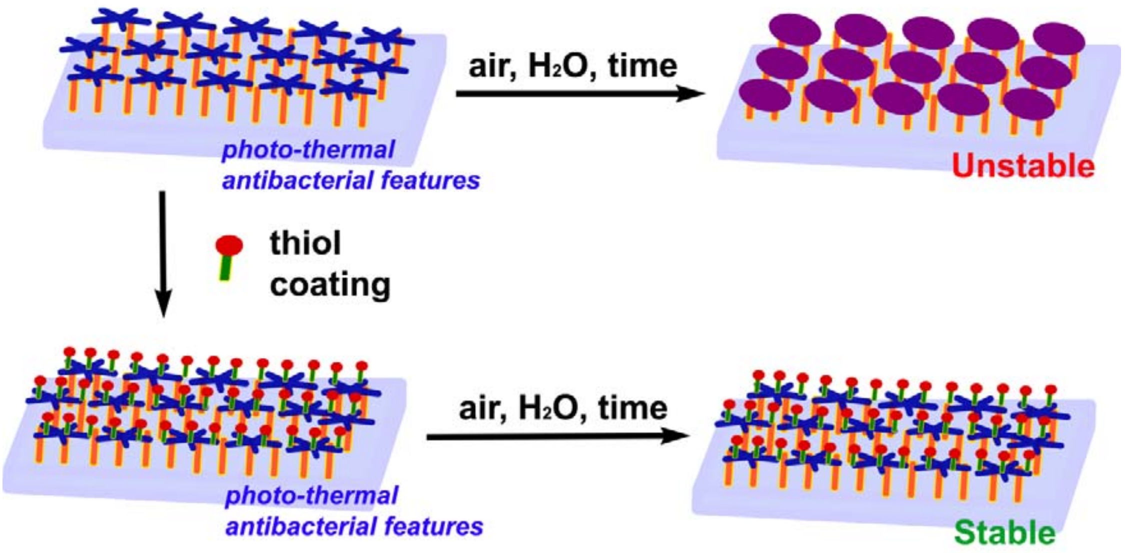

Figure S6. Schematic illustration of the improved stability obtained with thiol coating described in the work

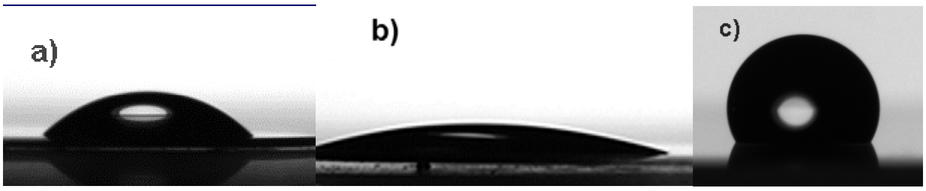

Figure S7. Example of contact angle images for: a) uncoated GNS monolayer, b) GNS monolayer coated with 4-MBA, c) GNS monolayer coated with 12C-SH
